# How Social Connectedness Helps Patients Stay Home After Hospital at Home Enrollment: A Mixed Methods Study

**DOI:** 10.1007/s11606-024-08785-9

**Published:** 2024-05-09

**Authors:** Christy J. W. Ledford, Lauren A. Cafferty, Eunice Lee, Hailie C. Hayes, Destine C. Ede, Brandon P. Hodges, Grant C. Whitebloom, David W. Walsh, Thad Wilkins

**Affiliations:** 1 Medical College of Georgia at Augusta University, Augusta, USA; 2 The George Washington University, Washington, DC USA

**Keywords:** Hospital-at-home, Care transitions, Social support

## Abstract

**Background:**

While enrolled in Hospital at Home (HaH) programs, patients rely on their social network to provide supportive behaviors that are routinely provided by hospital staff in the inpatient setting.

**Objective:**

This study investigated how social connectedness is associated with patient outcomes in a HaH program.

**Design:**

The explanatory iterative sequential mixed methods design included an electronic health record review to collect quantitative measures to describe the severity of patient illness and healthcare utilization and then qualitative interviews to explain quantitative findings.

**Participants:**

The quantitative phase included 100 patients (18 years or older) admitted to the hospital who were subsequently enrolled in the HaH program. In the qualitative phase, 33 of the 100 patients participated in semi-structured interviews.

**Analysis:**

Qualitative data was analyzed using the Sort & Sift, Think & Shift method. Integrated analysis included merged data displays of healthcare utilization data and patient descriptions of their care and genogram-type illustrations to enable variable-oriented analysis of structural support. We then examined patient narratives by two variables: life course and care elevation, to understand differences in the trajectories of six subsets of patients as identified by the quantitative data.

**Key Results:**

Three factors prompted patients to enroll in HaH: *low attention from hospital staff during hospital stay*; *loneliness and isolation during hospital stay*; and *family encouragement to enroll*. After discharge, social support within the home structure facilitated recovery during HaH. Conversely, HaH patients with limited support within the home were more likely to be readmitted.

**Conclusions:**

Structural social connectedness facilitates patient recovery in HaH. Before enrolling patients in HaH, clinicians should take an in-depth social history, including questions about social/familial roles, household responsibilities, and technology acceptance. Clinicians should engage formal and informal caregivers in these conversations early and communicate a clear picture of what caregivers should do to support the patient through recovery.

**Supplementary Information:**

The online version contains supplementary material available at 10.1007/s11606-024-08785-9.

## INTRODUCTION

Hospital at Home (HaH) programs provide hospital-level care within patients’ homes for a limited period of time as an alternative to hospital stays.^1,2^ HaH programs enable hospitals to accelerate the discharge of admitted patients by providing hospital-like services within the patients’ homes. While mortality and safety outcomes associated with HaH are similar to inpatient stays,^3,4^ patients in HaH programs are less sedentary and readmitted less frequently.^5^ Patients treated at home also report home environments that promote sleep and physical activity as well as better patient experience with the care team.^6^ HaH programs also benefit public health, increasing system-wide availability of hospital care, even during COVID-19 surges.^7^

While enrolled in HaH programs, patients rely on their social network to help them enact care supportive behaviors that are routinely provided by hospital staff in the inpatient setting.^8^ People in a patient’s social network become informal caregivers, altering their role and relationship with the patient.^2^ Thus, successful recovery may be associated with a patient’s social connectedness. Structural social connectedness is the structure and size of a social network, including whether individuals live with others or have regular contact with others.^9^ Furthermore, a person’s sense of the sufficiency of support from their social network is perceived as social connectedness.^9^ Both a person’s structural social connectedness and perceived social connectedness may influence their care and recovery in a HaH program as people in their social network provide instrumental support. Instrumental support is task-related assistance, including tasks associated with independent living, such as personal hygiene, cooking, and household chores.^10^

Using mixed methods, the present study investigated how social connectedness is associated with patient outcomes in a HaH program.

## METHODS

Our HaH model used a well-described early transition to home program^1,2^ where patients were admitted after an inpatient stay, usually two to five days. Resources included one daily telehealth visit from a physician or advanced practice provider, a daily telephonic visit from a registered nurse, remote vital sign monitoring three times daily and as needed, oral medications and supplemental oxygen as needed, and home health physical and occupational therapy. Patients communicated with the HaH team via telephone or encrypted audio/video and had continuous access to nurses and physicians for urgent issues. When patients did not respond to telephone calls, missed telehealth encounters, or failed to record vital signs, the team initiated welfare checks. Patients remained enrolled in HaH at the team’s discretion until discharged back to primary care. Emergency services were activated if escalation of care was needed to return patients to the physical hospital.

The study was an explanatory iterative sequential mixed methods design. In the quantitative phase, electronic health record review collected measures to describe the severity of patient illness and healthcare utilization. The qualitative phase followed the quantitative results to explain findings from the record review. Longitudinal mixed methods analysis enabled a holistic view of how multiple factors operate together in the lives of patients.^11^ The research team included one mixed methods expert, one qualitative expert, one hospitalist, one family physician, and six research assistants. The Augusta University Institutional Review Board Committee A determined this project as exempt from IRB review according to federal regulations, 45 CFR 46 (DHHS) 2018 Requirements. The authors adhered to the Consolidated Criteria for Reporting Qualitative Research (COREQ) [cite] to ensure comprehensive reporting of the qualitative phase of the study.

The present study was part of a larger study that conducted a matched retrospective chart review of 100 patients (18 years or older) admitted to the hospital who were subsequently enrolled in the HaH program from February 1, 2021, to January 31, 2022, matched to an inpatient stay cohort on age, sex, and illness severity (defined as admission to Intensive Care Unit). Results showed no difference in readmissions, time to readmission, or return emergency department visits between the HaH and inpatient stay cohort.^12^

The first and second authors (CL, LC), experts in mixed methods and qualitative methods, created the interview guide after the quantitative analysis was complete. The guide focused on HaH admissions process and home experience. See Appendix 1 for the interview guide. The first author trained four interviewers (HH, DE, BH, GW) in interview techniques and the interview guide.

In September 2022, interviewers attempted to contact the 100 HaH patients from the chart review via telephone to invite them to participate. Interviewers did not have a prior relationship with any of the interviewees. During the consent process, interviewers described the study’s purpose and confidentiality procedures. Interviews occurred during normal working hours. All participants consented to audio recording. Files were labeled by participant-selected pseudonyms. Audio files were transcribed. Identifiers were removed during a quality check of the transcripts. Following interviews, researchers met for structured debriefs that included discussion about the logistics of interviews and emerging topics of interest. Researchers wrote field memos during these meetings.

Qualitative data was analyzed using the Sort & Sift, Think & Shift method.^13^ This iterative process allows researchers to “dive in” the data to uncover dimensions and properties and “step back” to incorporate findings with existing literature. LC immersed herself in the data by reading through the transcript and field memos of each case to understand content and identify categories. She then stepped back to assess and identify important ideas.

**Figure 1:**
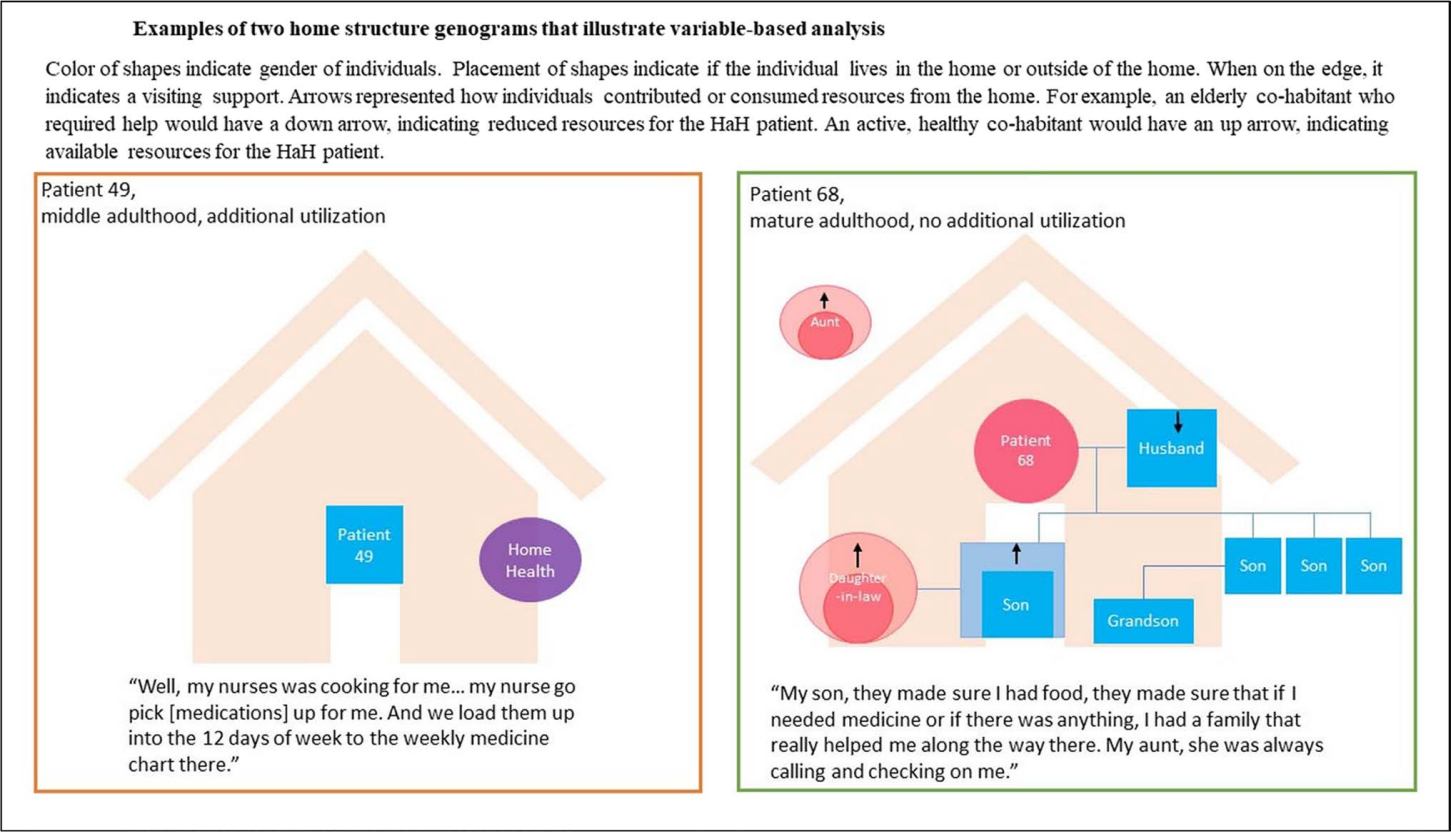
Examples of two home structure genograms that illustrate variable-based analysis. Color of shapes indicates the gender of individuals. Placement of shapes indicates if the individual lives in the home or outside of the home. When on the edge, it indicates a visiting support. Arrows represented how individuals contributed or consumed resources from the home. For example, an elderly co-habitant who required help would have a down arrow, indicating reduced resources for the HaH patient. An active, healthy co-habitant would have an up arrow, indicating available resources for the HaH patient.

The third author (EL) created merged data displays of quantitative healthcare utilization data and patient descriptions of their care timelines to conduct an intensive study of healthcare utilization for each patient. From the interview data, EL created genogram-type illustrations to enable variable-oriented analysis of structural support. See Fig. 1. Genograms depicted each patient’s family members (and other co-habitants) as well as formal and informal caregivers, indicating if they lived in the patient’s home before their enrollment in the HaH program and during the program. Interview data informed whether the individuals contributed or consumed resources from the home. Through review of these variables across genograms, LC, CL, and EL identified common topics occurring across cases.

In variable-oriented analyses, results of quantitative and qualitative phases were integrated during the discussion of outcomes of the entire study.^14^ In this analysis, we identified life-course-related factors that influenced patient outcomes and experiences, e.g., marriage, ages of children, and living parents. We then examined patient narratives by two variables: life course and care elevation, to understand differences in the trajectories of six subsets of patients as identified by the quantitative data. Life course was defined by middle adulthood, mature adulthood, and late adulthood.^15^ Middle adulthood (26–49 years old) was characterized by independence, potential marriage and/or children in the home, and career development. Mature adulthood (50–64 years old) was characterized by independence, marital transitions, children leaving the home, grandparenthood, and maturing careers. Late adulthood (65 and older) was characterized by retirement and death of peers. Care elevation was categorized as required additional acute healthcare utilization (through readmission or emergency department visit within 30 days of HaH enrollment) or did not require additional utilization.

This mixed methods analysis demonstrated not only which patients experienced recovery or readmission but also how social connectedness converged in a patient’s life to influence recovery and additional healthcare utilization needs.

## RESULTS

Of 100 HaH patients, 45 were successfully contacted. Of these, 33 (73.3%) completed interviews, for 621 min of recorded data or 259 pages of transcribed text. Table 1 presents individual characteristics. The analysis detected no significant differences between interview participants and non-participants. Table 2 presents quantitative measures collected from the electronic health record, including the 4C Mortality Score^16^ at the time of admission, length of stay in the HaH program, and whether the patient was readmitted or visited the emergency department within 30 days of enrollment.

**Table 1:** Individual Characteristics of Interview Participants (*n* = 33)

Age ^a^ at time of Hospital at Home enrollment		Mean 54.06 (sd 12.01)
Biological sex ^a^	Male	13 (39.4%)
	Female	20 (60.6%)
Racial and ethnic identity ^b, c^	Hispanic White American	1 (3%)
	Non-Hispanic Asian American	2 (6.1%)
	Non-Hispanic Black American	8 (24.2%)
	Non-Hispanic White American	21 (63.6%)
	Multiple racial/ethnic identities	1 (3%)
Marital status^a^	Single	4 (12.1%)
	Married	21 (63.6%)
	Divorced	3 (9.1%)
	Separated	1 (3%)
	Widowed	4 (12.1%)
Primary insurance^a^	Commercial	15 (45.5%)
	Medicaid	9 (27.3%)
	Medicare	5 (15.2%)
	Self-pay	4 (12.1%)

^a^ Measure collected from electronic health record

^b^ Racial and ethnic identity was collected as a structured, open-ended interview question: “When thinking about race or ethnicity, how do you describe yourself?” Qualitative data was coded and aligned with US Census language. Hispanic White Americans include patients who described themselves as Hispanic. Non-Hispanic Asian Americans include patients who describe themselves as Asian. Non-Hispanic Black Americans include patients who described themselves as African American, Afro-American, Black, or Hebrew Israelite. Non-Hispanic White Americans include patients who described themselves as Caucasian or White. Multiple racial/ethnic identities include one patient who described themselves as mixed

^c^ In the larger study, the HaH patients and usual care patients differed significantly in terms of race (p < 0.001). More HaH patients were non-Hispanic White Americans, and more usual care patients were non-Hispanic Black Americans

**Table 2:** Health Measures^a^ of Interview Participants (*n* = 33)

4C Mortality Score^16^ at time of admission		Mean 6.85 (sd 2.67)
Length of stay in Hospital at Home program (in days)		Mean 7.24 (sd 5.04)
Care elevation	Inpatient readmission or emergency department visit within 30 days	5 (15.2%)
	No inpatient readmission or emergency department visit within 30 days	28 (84.8%)

^a^ Measure collected from electronic health record

In integrated analysis, we identified two primary lines of inquiry regarding social connectedness. First, three social network factors influenced patient decision-making in the enrollment process. Second, after enrollment in the HaH program, three factors influenced patient recovery.

### Social Connectedness in Enrollment

Social network factors influenced patient decision-making in the enrollment process. Three factors prompted patients to enroll in HaH: *perception of low attention from hospital staff during their admission*; *loneliness and isolation linked to visitor limitations*; and *family encouragement to enroll*.

Patient experiences during the hospital stay influenced decisions to enroll in HaH. Hospital experiences were inseparable from the system burdens of the pandemic. Patient descriptions reflected multiple spaces throughout the hospital, including interactions in the emergency department, intensive care unit, and on the floor. Participant 17 (mature adulthood, no additional utilization) connected the overloaded system with HaH enrollment. He said, “One of the reasons for the at-home program was so that I could get out of the hospital for my benefit, but at the same time, I believe the hospital was kind of overloaded.”

During their hospital stay, patients described loneliness and isolation. The pandemic amplified this experience when visitor restrictions were implemented. Participant 25 (mature adulthood, no additional utilization) compared the pain of isolation to her physical symptoms: “The isolation was every bit as hard as not being able to breathe.” Participant 51 (middle adulthood, no additional utilization) communicated the gravity of being alone, “I kept paging them, asking them can I go home,’cause I felt so alone, I felt lonely, and I was in that dark room all alone…[they said] we’ll see if we can get you in that at-home program.” Patients described that HaH provided them with an alternative to the isolating hospital environment. Participant 15 (late adulthood, no additional utilization) explained, “My husband couldn’t come [visit in the hospital]. Nobody could come see you … [HaH allowed me] to go home.”

Patients did not make the decision to enroll alone. Patients described how their family and caregivers influenced the decision. Although family members wanted patients to return home, they also recognized patients’ complex needs and questioned their ability to provide adequate support. For example, Participant 74’s (mature adulthood, additional utilization) wife, who was also the primary caregiver for her elderly mother, encouraged Participant 74 to enroll in the program for extra monitoring. Participant 74 confirmed, “I really did it for my wife, because there was no way she was going to be able to tend to both me and her mother.” Participant 74 recognized that he needed quick access to care. He remembered that “‘You’ll have a team virtually 24/7 if you need them’ and I think that was the selling point for my wife…if something goes wrong, he’s got somebody.”

### Social Connectedness in Recovery

Once enrolled in the HaH program, patients described three factors that influenced their recovery: *home as a comfortable place*, *instrumental support required*, and *instrumental support availability in the home.* Integrated analysis revealed that the five patients who were readmitted or visited the emergency department within 30 days of enrollment all described limited instrumental support available in the home.

Home was a comfortable place that facilitated independence and the ability to be with loved ones. Participant 52 (mature adulthood, no additional utilization) described, “It’s a wonderful tool to be in your own bed, your own sheets, your own shower. You have a little bit of autonomy.” Similarly, Participant 100 (late adulthood, no additional utilization) explained the freedom she experienced: “It was peaceful, and I could do what I wanted to do when I wanted to do with it when I was able. I could get up and eat or go outside and sit in the sunshine.”

Patients recognized how social support in the home aided in their recovery. Participant 28 (mature adulthood, no additional utilization) said, “I did not have a good mental state when I was in the hospital because of the fears and the inability to have that support surround me…being at home helped my recovery time. It didn’t take as long for me to recover because I did have that support [at home].”

Recovering at home required instrumental support. Some participants described strong social support, within and outside the home structure, that facilitated activities of daily living (ADLs). Participant 5 (mature adulthood, no additional utilization) explained, “I had a good support system because my daughter and my son-in-law…we live about a mile apart…having my daughter a nurse and my husband also a nurse… we even had neighbors, so I knew if I needed anything, I had someone to help.”

As described by patients, the most essential instrumental support needed was food. Participant 22 (middle adulthood, no additional utilization) described, “It helped me being home, because my family could just drop off food at the house for me or [they could] cook what I wanted, so I could get nutrition that I needed.” Participant 51 (middle adulthood, no additional utilization) described how food was just one way her family facilitated ADLs. She said, “If I was hungry or anything, [my family] would bring [food] to me, help me up out of bed sometimes, walk me outside, help me get back in, basically help me take a shower and bathe like that until I got better.” When their basic needs were supported, patients shifted their focus toward medical recovery.

Supportive others also helped with medication administration and health care. Participant 100 said, “[My daughter] was the one that had my medicine lined up to give me at a certain time and wake me up with a phone call when it was time for the doctor to call. She takes me wherever I need to go to see a doctor or anything…I couldn’t have done it without her.”

In contrast, patients who were themselves caregivers described challenges that interfered with recovery. Some patients were caregivers for younger (i.e., children), disabled, or elderly family members. Participant 46 (mature adulthood, no additional utilization), who was the primary caregiver for her disabled son, recounted, “It was a nightmare to make an omelet for my son to give him something to eat for a day, so food was always a serious problem and nobody to get it…I got older and older day by day…I [unintentionally] lost probably close to 20 pounds.”

Patients across life course stages recalled that at enrollment, they perceived they had a network of people they could rely on for instrumental support, such as extended family, friends, or home health services. However, when support networks could not satisfy the demand of instrumental needs, patient recovery was hindered. Participant 20 (middle adulthood, additional utilization) said she had friends who provided meals, picked up prescriptions, and drove her to appointments. However, she struggled to manage HaH computer-mediated self-management tasks alone. She explained, “As far as doing my vitals, all that was pretty much mainly me.” As she experienced technological challenges with program equipment and processes, it added stress rather than facilitated her recovery. “The number of phone calls, it was just crazy…you’re having to talk to somebody four or five times a day about [device] not working, and that’s harassing.” For Participant 20, the HaH experience was more tiresome than her previous hospital stays.

Patients with limited support inside the home also described how they realized with the severity of their illness they could have received better care in the hospital. Participant 62 (mature adulthood, additional utilization), a single mother living with her son, said, “Just walking down the hallway from my bedroom to the living room I was so out of breath…I would have rather stayed in the hospital. I was just too far gone with the COVID.”

When patients did not have cohabitating others who could provide around-the-clock attention, their recovery slowed. Although Participant 49 (middle adulthood, additional utilization) received instrumental support through private home health nurses, they were unable to provide the comprehensive support he needed. Living alone, he struggled: “I had different [nurses] call to check on me at night. My nurses was cooking for me…, but I [had difficulties with taking a bath or getting dressed]. I was very weak.”

Patients described how having another person living in their home did not consistently confer support for their health needs. Patients described how household members had competing needs with the patients’ caregiving needs. Participant 76 said, “My youngest at the time wasn't able to drive, so it was hard for her to get her to extracurricular stuff…My oldest was working at the time so it was hard for her to be able to help…” Participant 74’s (mature adulthood, additional utilization) in-home support was also disrupted by the pandemic. He recalled, “[Wife] was working 12-h days, sometimes six days a week.” With his wife unavailable to help, he resisted asking for help from supportive others outside the home. Participant 74 explained, “Not because nobody didn’t want to help. I just wanted to be left alone so I could do what I needed to do.”

## DISCUSSION

This mixed methods study reveals how social connectedness influences patient decisions to enroll in a HaH program and how social connectedness in the home influences their recovery. At enrollment, patients considered the attention they received from hospital staff, their experiences of isolation in the hospital, and how their families and friends encouraged participation in the HaH program. To optimize health outcomes in the HaH program, patients needed structural social connectedness within their home that was comparable to the instrumental support they would have received in a hospital stay. The study reveals the importance of shared decision-making^17^ among the patient, their family (formal and informal caregivers), and the healthcare team before enrollment in a HaH program to improve patient health and wellbeing.

Perceived and structural social connectedness influenced patient experiences in a HaH program. Social support within the home structure facilitated recovery within the HaH program. Conversely, HaH patients with limited support within the home structure were more likely to be readmitted. These findings align with previous studies on general hospital discharge that demonstrated that social support can decrease illness severity and reduce recurrent hospitalizations.^18,19^ While HaH patients benefited from general social support, they needed substantial instrumental support that was available around the clock, just as it would be in an inpatient hospital setting. Patients who struggled with accomplishing ADLs alone were more likely to need additional care, which aligns with other research showing that difficulty performing ADLs contributes to readmission.^19^

Perceived social connectedness and direct social influence also impacted decisions to enroll in the HaH program. Previous research demonstrated that lack of social support at home was associated with declinations to participate in a HaH program.^20^ Here, patients also relied on supportive others in these decisions. In our study, at admission into the HaH program, patients stated they had friends and family members to provide support, but oftentimes, it was limited to errands outside of the home and not continuous support with ADLs. Previous studies on hospital discharge emphasized the importance of clinician conversations of potential social support limitations^21^ and that the screening (qualifying) questions about patient support at home must be more open-ended than a simple yes/no.^21^

Moreover, when the healthcare team asks about patient support during conversations at enrollment, patients may not understand that their answer to this question is an important determinant of the success in the program. They may assume that the healthcare team has decided already that they are a good candidate for the program without knowing the scope of their social support. Patients likely do not have a clear understanding of the amount of instrumental support they need. Caregivers play a critical role in patients’ successful transition to home after a hospital stay.^22^ This role is expanded in HaH programs, in which the patient is still in active recovery. Previous quantitative research with family members showed that they did not feel that HaH programs shifted the burden of care from hospital staff to themselves.^23^ However, our findings show that patients routinely needed basic and complex instrumental support from caregivers to enact HaH processes.

Findings should be considered in the context of three specific limitations. First, this study was conducted in a specific epoch of time. The viral nature and uncertainty surrounding COVID impacted hospital and home medical decisions through 2021 and 2022.^7,24–27^ Part of that context was strict hospital visitation rules, which increased patient distress.^28^ Previous research from the pandemic demonstrated that patients were motivated to “escap[e] from hospital routines” to home.^29^ These restrictions motivated patients in our sample to enroll in HaH. However, stricter hospital visitation rules are not unique to COVID,^30^ and this motivation should be considered in all patient screening for HaH. COVID also reduced patients’ capacity to recover at home as patients limited their exposure to supportive others. Again, this should be part of the screening conversation as clinicians help patients think through if their medical condition requires additional infection control at home. Second, the language of a HaH program can be confusing for patients. In interviews, some patients explained HaH services as part of a larger self-management landscape that included primary care chronic care management, home health workers, and other telemedicine programs. Third, interviews focused on the patient describing their experiences of severe illness. Some interviewees commented on how they could not remember the number of days they were in the program. However, they were confident in their descriptions of needs and support through their illness. In analysis, we triangulated patient interview data with electronic health record information, but patients’ overall perceptions of a single hospital program are likely inseparable from their overall healthcare experience in a certain time period.

As hospitals increasingly implement HaH programs, clinicians need to strategically screen patients for participation in the programs. As clinicians screen patients for HaH enrollment, they should conduct an in-depth social history, using a tool such as the D-CEGRM social resource interview.^31^ Questions should include social and familial roles and household responsibilities. Screening should also include questions about technology acceptance^32^ and use, and ehealth literacy^33,34^ to ensure that patients or formal and informal caregivers can adequately utilize the technology required for HaH. It is important to consider the patient’s practical and emotional support system when referring them to a HaH program. Serious illness itself is a stressor, and potential isolation without social support can lead to depression, resulting in readmission.^19,35^ The decision to enroll in a HaH program should include shared decision-making among the patient, their formal and informal caregivers, and the healthcare team.^17^ Clinicians should engage formal and informal caregivers in these conversations from the start, ensuring they understand what instrumental support will be needed. This is especially critical when caregivers are the persons who will provide instrumental support. Clinicians should communicate a clear picture of what caregivers will do to support the patient through recovery.

## Electronic Supplementary Material

Below is the link to the electronic supplementary material.

Supplementary file1 (PDF 358 KB)

## Appendix 1. Interview Guide

Tell me about what brought you to the hospital.

What was your experience like when you were at the hospital?

What motivated you to sign up for hospital at home?

Tell me about your living situation during the program. (Including probes, such as… How many people were staying with you [or living in your home] during the program?, What was your relationship with them?).

Tell me about what a day looked like for you in the program. (Including probes, such as… How did your family or friends support you during this time?, How did your family or household roles or schedules change during the experience?).

How did being at home change the quality of care you received?

How would you change the hospital at home program for patients like you?

What advice would you give patients considering the program?

What more would you like to share about your experience?
